# Psychometric Validity of the Anxiety Inventory for Respiratory Disease (AIR) Scale in an Indian Sample of Chronic Obstructive Pulmonary Disease: A Cross-Sectional Study

**DOI:** 10.7759/cureus.36241

**Published:** 2023-03-16

**Authors:** Prabhoo Dayal, Mahendra S Uikey

**Affiliations:** 1 National Drug Dependence Treatment Centre, All India Institute of Medical Sciences, New Delhi, New Delhi, IND; 2 Psychiatry, All India Institute of Medical Sciences, New Delhi, New Delhi, IND

**Keywords:** mini-international neuropsychiatric interview (m.i.n.i.), anxiety and psychosomatic symptoms in chronic diseases, dsm 5, copd: chronic obstructive pulmonary disease, concurrent criterion validity

## Abstract

Introduction

Researchers have found that chronic obstructive pulmonary disease (COPD) patients suffer from anxiety more than the general population. The Anxiety Inventory for Respiratory Disease (AIR) scale has been primarily used to assess non-somatic anxiety in COPD patients. The validity of AIR among COPD patients has not been evaluated in Indian settings. Therefore, this study was undertaken to determine the validity of AIR in these patients. The study aimed to evaluate concurrent criterion and discriminative validity of the AIR screening scale among patients with COPD using Mini International Neuropsychiatric Interview (MINI) 7.0.2 as the gold standard measure for diagnosing Diagnostic and Statistical Manual of Mental Disorders, Fifth Edition (DSM-5) anxiety disorders.

Materials and methods

A cross-sectional study was conducted in the Outpatients Department (OPD) of the Department of Pulmonary Medicine, All India Institute of Medical Sciences (AIIMS), New Delhi, from August 2018 to July 2019. A total of 100 patients diagnosed with COPD and aged 30 or above were recruited. All participants were further assessed in person by a psychiatry resident doctor using semi-structured proforma, MINI 7.0.2, and AIR Disease (Hindi). Mann-Whitney U and receiver operating characteristic (ROC) curves were conducted. The two-sided p-value of less than 0.05 was considered to be statistically significant.

Results

To assess the concurrent criterion validity of the AIR scale for screening clinical anxiety disorders, the ROC curve was constructed using MINI diagnoses of anxiety disorder as the gold standard measure. A cut-off score of 5.5 was found to maximize both the specificity and sensitivity of the AIR scale for screening anxiety disorders among COPD patients with COPD. The AIR scale showed a high sensitivity (95%) and specificity (89%) at this cut-point.

Conclusion

The findings of this study recommend a cut-off score of 5.5 on the AIR scale instead of 8 in previous studies, as maintaining the previously recommended cut-offs in Indian settings may lead to an increase in false negatives. This could have negative consequences for patients seeking treatment. Further studies may be planned to explore the psychometric properties of the current tool in a larger population.

## Introduction

Large-scale epidemiological studies have reported that chronic obstructive pulmonary disease (COPD) is globally prevalent at 10.3% [1]. The prevalence of anxiety varies from 10 to 55% in patients with COPD [2,3]. Literature shows that comorbid anxiety symptoms may lead to poor quality of life, frequent exacerbations, increased number of re-hospitalizations, and health care utilization [4-7]. Various studies suggest that routine screening for depression and anxiety in COPD patients and an integrated treatment approach may improve the treatment outcomes. Unfortunately, anxiety symptoms may go undetected in routine clinical practice due to factors such as physician-perceived barriers, system-related barriers [8,9], and difficulty in differentiating these anxiety symptoms from somatic symptoms of COPD like breathlessness. Therefore more than 50% of COPD patients with comorbid anxiety are not getting any appropriate treatment [8].

Various studies have used Hospital and Anxiety Depression Scale (HADS) and Becks Depression Inventory (BDI) in patients with COPD but these screening tools are mainly validated for other chronic medical illnesses patients [10]. Some of the items in these screening tools are based on somatic symptoms that may affect the scoring and diagnosis of anxiety disorder because of some overlapping symptoms of COPD and anxiety disorders. The Anxiety Inventory for Respiratory Disease (AIR) scale [11] has been primarily used to assess non-somatic anxiety and lacks somatic symptoms-based questions for screening anxiety in COPD patients, although previous studies have shown variable cut-off scores of AIR for diagnosing clinical anxiety in COPD patients.

AIR has not yet been evaluated for appropriate cut-off scores in Indian settings for COPD patients, even though Yohannes and Willgoss et al. [12] recommended that a threshold score of ≥8 on the AIR scale may be utilized for screening anxiety disorders in patients with COPD. The study aimed to evaluate concurrent criterion and discriminative validity of the AIR screening scale among patients with COPD using Mini International Neuropsychiatric Interview (MINI) 7.0.2 as the gold standard measure for diagnosing Diagnostic and Statistical Manual of Mental Disorders, Fifth Edition (DSM-5) anxiety disorders.

## Materials and methods

1.1 Type of study

A cross-sectional study was conducted in the Outpatients Department (OPD) of the Department of Pulmonary Medicine, All India Institute of Medical Sciences (AIIMS), New Delhi, from August 2018 to July 2019. A total of 100 patients aged 30 and above, who were diagnosed with COPD, were recruited through the purposive sampling approach.

1.2 Study subjects and eligibility

Patients with COPD undergoing treatment in the Department of Pulmonary Medicine, AIIMS, New Delhi, India meeting the inclusion criteria and showing their interest in participation were included after obtaining their informed consent. The inclusion criteria for the study were male COPD patients aged 30 or above, and their willingness to participate in the study. The study exclusion criteria were patients with comorbid medical illnesses (congestive heart failure, diabetes mellitus, and cerebrovascular accident), those who were taking oral corticosteroids, and patients who did not agree to participate. Ethical approval was received from the Institute Ethics Committee (Ref. No. IECPG-310/18.07.2018). Written informed consent was obtained from all study participants.

1.3 Translation of the Anxiety Inventory for Respiratory disease (AIR) scale

The Hindi translation of the English version of the AIR scale was carried out with the permission of the original authors using guidelines recommended by the American Association of Orthopedic Surgeons [13,14]. First, two qualified experts (Pulmonologist (T1) and Psychiatrist (T2)) translated the original English AIR scale into Hindi. Though, their mother tongue was Hindi but had a good command of English as well. The translators tried to use simple words in the Hindi language considering the general population as respondents and to achieve cross-cultural and conceptual equivalence of AIR in Hindi. In this step, the corresponding author along with both translators, T1 and T2, compared both translated versions and produced one common consensus translation T12. Then two independent translators with good command over English translated T12 Hindi translated scale back to English. They were blind to the original AIR scale. The conceptual and cultural equivalence was focused upon in spite of equivalence during the process of back-translation and this was repeated until a satisfactory and final version is reached [15]. Finally, an expert committee comprising researchers, mental health professionals, pulmonologists, and all the translators was constituted to identify the issues related to concepts of translation and any differences between the English version of AIR and Hindi translation. All the members of this constituted committee were Hindi-English bilinguals. The committee of the expert panel coherently integrated all the versions of the scale to synthesize the pre-final version of the AIR scale into Hindi. In the last stage, 10 patients were interviewed having COPD by using the pre-final synthesized AIR-Hindi scale. For each item, details of the patients’ opinions and their interpretations were collected along with their responses. Responses and opinions were analyzed to check for any syntax or grammatical errors and misunderstood vocabulary in the translation. Necessary changes were made for synthesizing the final Hindi version of the AIR scale. These interviews were conducted by two psychiatrists.

1.4 Sample size (100)

As per the COSMIN (COnsensus-based Standards for the selection of health Measurement INstruments) study design checklist [16], a sample of at least 100 participants is considered good for exploring measurement properties. The sample size of 100 COPD patients provided more than 90% power for the detection of a difference of 0.25 between receivers operating characteristic (ROC) areas under the curve (AUCs) along with a two-sided type I error rate of 5%.

1.5 Measures

For this study, data from all measures used in the MD psychiatry thesis were available. Before all analyses, hypotheses were generated based on the available measures used in the MD psychiatry thesis. We used only the results of measures relevant to the instrument validation study.

The Hindi-translated AIR was utilized as a screening tool for screening out anxiety symptoms in COPD patients. MINI 7.0.2 was used as a "Gold Standard" diagnostic measure to increase diagnostic accuracy for anxiety disorders in COPD patients. During in-person visits, the MINI interview was performed by a trained MD Psychiatry final year resident doctor to confirm the presence of any anxiety disorder.

1.5.1 Socio-demographic and Clinical Characteristics

In the COPD outpatient clinic, each patient had a proper medical record including socio-demographic characteristics, medical history, results of physical examination, lab test results, pulmonary function test (PFT), and COPD assessment records. Age, education level, marital status, occupation, residential area, COPD status, other medical co-morbidities, and current smoking status were recorded.

1.5.2 Anxiety Inventory for Respiratory Disease (AIR)

The AIR [11] scale consists of 10 items for screening clinical anxiety. The total score can be calculated by using a 4-point scale that ranges from 0 ("not at all") to 3 ("nearly all the time") and lies within the range of 0 to 30. If the score obtained on the AIR is at least 8 or higher then it indicates clinically significant anxiety symptoms.

1.5.3 Mini-International Neuropsychiatric Interview, Version 7.0.2

Anxiety disorders were assessed using the MINI, version 7.0.2 [16] which included panic disorder, agoraphobia, social anxiety disorder, PTSD, social phobia, and generalized anxiety disorder. With an exception of panic disorder, all the above-rated disorders have current symptoms. The only panic disorder includes present and lifetime/chronic symptoms. For the existence of any anxiety disorder, a composite score was calculated.

2.1 Statistical analysis

To summarize the socio-demographic and clinical features of the sample, as well as the scores for the scale, descriptive statistics were used. Categorical data were presented in terms of numbers and frequencies of observations, whereas continuous data as means ± standard deviation or median if the data did not follow a normal distribution.

To examine the discriminant validity, a two-tailed independent t-test/Mann-Whitney U was used for comparing AIR Hindi scores divided into two sets based on the presence or absence of DSM-5 for any anxiety disorder on MINI. To assess the criterion validity of AIR Hindi for screening clinical anxiety disorders, the ROC curve was constructed using MINI diagnoses of anxiety disorder as the gold standard measure, and the sensitivity and specificity were evaluated to obtain the optimal cutoff score. Youden’s J (=(sensitivity + specificity) - 1) was calculated to compute the optimal cut-off value positive predictive value (PPV), and negative predictive value (NPV) was also analyzed. All statistical tests were two-tailed, and p-values below 0.05 were considered statistically significant. The data analysis was carried out by Statistical Product and Service Solutions (SPSS) (IBM SPSS Statistics for Windows, Version 22.0, Armonk, NY).

## Results

This study enrolled 100 patients with COPD and identified 18% of them as having any anxiety disorder on MINI.

3.1 Discriminative validity

Mean scores of AIR (Hindi) in participants with any anxiety disorder diagnosis on MINI were compared to mean scores of those without any anxiety disorder diagnosis on MINI using the Mann-Whitntey U Test. There was a significant difference in mean scores of AIR (Hindi) between those with any anxiety disorder on MINI and those without (U=68, P<0.001). No significant differences were there in age, education, occupation, COPD severity, or smoking status between those with and without any anxiety disorder on MINI (Table 1).

**Table 1:** Mean scores of AIR (Hindi) in participants with any anxiety disorder diagnosis on MINI were compared to mean scores of those without any anxiety disorder diagnosis on MINI using the Mann-Whitney U Test. MINI: Mini International Neuropsychiatric Interview; AIR: Anxiety Inventory for Respiratory Disease

Variables	Total (n=100)	Any anxiety disorder present on MINI (n=18)	Any anxiety disorder absent on MINI (n=82)	Value^#^
	Mean (SD)	Mean (SD)	Mean (SD)	
AIR (Hindi) Scores	3.27 (3.96)	1.93 (2.60)	9.39 (3.31)	U=68, P<.001

3.2 Concurrent criterion validity

**Figure 1:**
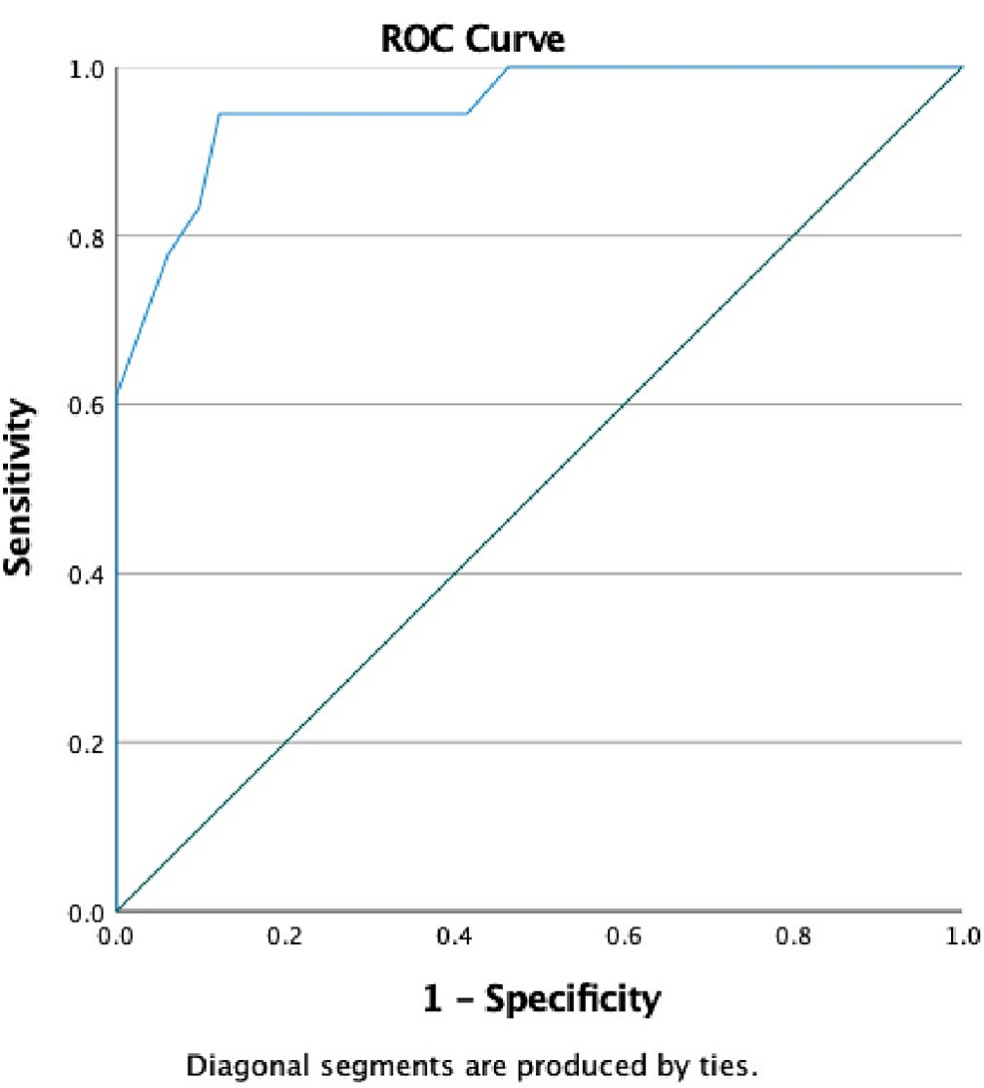
Receiver operating characteristic curves for Anxiety Inventory for Respiratory Disease (AIR) (Hindi) for correct identification of MINI diagnosis of any anxiety disorder. The area under the receiver operating characteristic (ROC) curve for Anxiety Inventory for Respiratory Disease (AIR) (Hindi) was 0.95 (95% CI, 0.90-1.00). MINI: Mini International Neuropsychiatric Interview

The ROC curve (ROC) for the AIR (Hindi) was constructed using MINI diagnoses of anxiety disorder as the gold standard. The AUC was 0.92 (95% CI, 0.96-1.00) (Figure 1).

For each AIR (Hindi) cutoff point sensitivity (true positive rate), specificity (true negative rate), +PPV, and -NPV were computed. Youden’s index ((sensitivity + specificity) - 1) was computed to find the highest sensitivity and specificity simultaneously. The score of 5.5 on AIR (Hindi) was taken as the optimal cutoff score; yielding a specificity of 0.85, a sensitivity of 0.93, a PPV of 93%, and an NPV of 98% (Table 2).

**Table 2:** For each AIR (Hindi) cutoff score sensitivity, specificity, positive predictive value (+PPV), and negative predictive value (-NPV) based on receiver operating characteristic (ROC) analysis. Bold numbers indicate the optimal cutoff points for AIR (Hindi) scale. NPV: negative predictive value; PPV: positive predictive value; AIR: Anxiety Inventory for Respiratory Disease. Youden’s index ((sensitivity + specificity) - 1) was computed to find the highest sensitivity and specificity simultaneously.

AIR Cutoff Score	Sensitivity	1-Specificity	Specificity	Youden’s index	PPV, %	NPV, %
3.50	.944	.244	0.756	0.73	45.9%	98.4%
4.50	.944	.207	0.793	0.737	50.0%	98.5%
5.50	.944	.122	0.878	0.822	63.0%	98.6%
6.50	.883	.098	0.902	0.785	65.2%	96.1%
7.50	.778	.061	0.939	0.778	73.7%	95.1%
8.50	.611	.000	1	0.611	100.0%	92.1%
9.50	.500	.000	1	0.5	100.0%	90.1%
10.50	.389	.000	1	0.389	-	-

## Discussion

This study used MINI, as the gold standard measure for diagnosing anxiety disorders, and validated the AIR. The sample size was as per the directions of the COSMIN study design checklist. The MINI was administered by an MD final-year student in psychiatry. In this study, a cut-off score of 5.5 was found to maximize both sensitivity and specificity of the AIR scale for screening anxiety disorders among COPD patients. A specificity of 0.85, a sensitivity of 0.93, NPV of 98%, and PPV of 93% were the yielded results (Table 2).

Authors of the original development and validation of AIR who used the Patient Health Questionnaire (PHQ) for concurrent validity reported a cutoff score of 14.5 with a sensitivity of 0.93 and specificity of 0.98 for detecting clinical anxiety. The higher cuff-off score of AIR in original the development study may be due to the use of the PHQ as a diagnostic tool which does not serve as a “gold standard” for the diagnosis of anxiety disorders [11].

MINI was used in a study to diagnose anxiety disorder in COPD patients, and a threshold score of 8 on the AIR scale was recommended as an acceptable cut-off for identifying anxiety disorders, with a sensitivity of 0.80, specificity of 0.75, and a PPV of 67% and NPV of 81% [12]. However, Baker et al. used MINI, as the gold standard for diagnosing anxiety disorders, and found that a cut-off score of 8 on AIR showed a sensitivity of 0.67, specificity of 0.65, PPV of 19%, and NPV of 94% [17]. This study has used MINI as a gold standard and administered by trained MD psychiatry residents. The findings from this study showed that 18% of patients had any anxiety disorder in COPD patients based on MINI. Also, this study has an adequate sample size of 100 participants with COPD. A lower threshold score of 5.5 rather than 8 on the AIR, maximized sensitivity and specificity simultaneously for screening anxiety disorders among COPD patients in this study (Table 2). The findings of this study recommend a cut-off score of 5.5 on AIR instead of 8 in previous studies, as maintaining the previously recommended cut-offs in Indian settings may lead to an increase in false negatives. This could have negative consequences for COPD patients seeking treatment.

4.1 Clinical implications

There are a variety of possible causes of anxiety symptoms; therefore, it is difficult to determine whether the symptoms are due to COPD itself or to psychiatric conditions occurring concurrently with COPD. COPD symptoms such as dyspnea and fatigue can also occur in mood or anxiety disorders. Additionally, the pharmacological treatment for COPD such as beta-agonist bronchodilators and corticosteroids can contribute to mood or anxiety symptoms. The AIR scale does not inquire about somatic symptoms which are often confused with the physical symptoms of COPD. In this study, a cut-off score of 5.5 was found to maximize both the sensitivity and specificity of the AIR for screening anxiety disorders among COPD patients. The AIR scale showed a high sensitivity (95%) and specificity (89%) at this cut-point. The AIR as an anxiety screening instrument demonstrated good psychometric features for identifying screening symptoms of clinical anxiety in COPD patients in Indian settings. This study indicates that optimum cuff-off scores for Indian settings differ from those recommended by the original authors, this may be due to cultural differences and other factors such as assessment instruments, sample size, and recruitment methods used in previous validation studies.

5.1 Limitations

This study, however, has its limitations. Specifically, it only studied male patients with COPD and included data from just one clinic site.

## Conclusions

The AIR as an anxiety screening instrument demonstrated good psychometric features for identifying screening symptoms of clinical anxiety in COPD patients in Asian settings. The findings of this study recommend a cut-off score of 5.5 on AIR instead of 8 in previous studies, as maintaining the previously recommended cut-offs in Indian settings may lead to an increase in false negatives. This could have negative consequences for COPD patients seeking treatment.
